# The journey from opposition to recovery from eating disorders: multidisciplinary model integrating narrative counseling and motivational interviewing in traditional approaches

**DOI:** 10.1186/2050-2974-1-19

**Published:** 2013-06-06

**Authors:** Moria Golan

**Affiliations:** 1 Shahaf, Community Services for the Management of Weight-Related Problems, Department of Nutrition, Tel Hai Academic College, Upper Galilee and School of Nutritional Sciences, the Hebrew University of Jerusalem, Jerusalem, Israel

**Keywords:** Anorexia nervosa, Bulimia nervosa, Transdiagnostic model

## Abstract

**Background:**

In the world of today’s of ever-briefer therapies and interventions, people often seem more interested in outcome than process. This paper focuses on the processes used by a multidisciplinary team in the journey from opposition to change to recovery from eating disorders. The approach outlined is most relevant to those with severe and enduring illness.

**Methods:**

This paper describes a five-phase journey from eating-disorder disability and back to health as it occurs for patients in a community-based facility. This integrative model uses narrative and motivational interviewing counseling weaved into traditional approaches. It approaches illness from a transdiagnostic orientation, addressing the dynamics and needs demanded by the comorbidities and at the same time responding effectively in a way that reduces the influence of the eating disorder.

The treatment described involves a five-phase journey: Preliminary phase (choosing a shelter of understanding); Phase 1: from partial recognition to full acknowledgment; Phase 2: from acknowledgment to clear cognitive stance against the eating disorder; Phase 3: towards clear stance against the “patient” status; Phase 4: towards re-authoring life and regaining self-agency; Phase 5: towards recovery and maintenance.

**Results:**

In a longitudinal study of patients with a severe and debilitating eating disorder treated with this approach. The drop-out rate was less than 10%. This was during the first two months of treatment for those diagnosed with bulimia nervosa, and this was higher than in those diagnosed with anorexia nervosa. At the end of treatment (15 months to 4 years later) 65% of those treated with anorexia nervosa and 81% of those treated with bulimia nervosa were either in a fully recovered state or in much improved. At the four-year follow-up, 68% of those diagnosed with anorexia nervosa and 83% of those diagnosed with bulimia nervosa were categorized as either fully recovered or much improved. All patients who completed the program were gainfully employed.

**Conclusions:**

The collaborative work, which is the heart of the described model increases the patient’s and family’s ownership of treatment and outcome and strengthen the therapeutic bond, thus enhances recovery.

## Introduction

The eating disorders Anorexia Nervosa (AN) and Bulimia Nervosa (BN) are severe psychiatric disorders that most commonly begin during adolescence; the teenage years form a critical period of significant changes in both biological and psychosocial development. Patients with eating disorders (EDs) often experience other psychiatric disorders. Axis I psychiatric disorders (including depression, anxiety, body dysmorphic disorder, or addiction disorders) and Axis II personality disorders (particularly borderline personality disorder) are frequently seen [1]. The characteristics of these concurrent conditions increase the complexity of treatment and necessitate additional counseling skills [1].

Ambivalence regarding recovery has been understood by the different theorists as expressions of selfless souls with difficulties in achieving self-regulation, calming, soothing, and vitalizing [2,3].

It may present defenses against high-reward dependence, arrested self-development [4-6], negative perceived reality due to ego weakness, anxiety, and interpersonal factors [7,8].

Resisting treatment is actually a considerable investment in the patient’s need to maintain control over his/her internal and external worlds and the objects within them [9], a way to remain immature, with no responsibilities, adoption of narrow views, and protection from life’s demands. Thus the ED is less about food and weight than about trying to protect the sufferer from facing social, familial, or personal pressures [10,11]. By refocusing one’s attention onto weight, shape, and eating, one gains a sense of emotional control and a sense of accomplishment. Moreover, by eating and/or by losing weight, patients attempt to reduce anxiety and elevate their mood, at least in the short term [12-14]. The ED thus provides a sense of achievement [15] and becomes a way of control [16]. Therefore, it is not surprising that most ED patients are ambivalent about change and the more severe ED patients are either in denial and/or opposition to change [17]. Pryor, Johnson, Wiederman, and Boswell have stated that “deniers” often maintain a sense of arrogance and superiority with respect to their anorexic symptoms. They seem to view themselves as superior to other people who are “weak” and “give in” to bodily needs and desires and undervalue the cost of the illness and the self-harm it causes [18]. Only a few empirical studies exist to allow a data-based examination of the proportion of patients who are ambivalent about recovery. Yet, Blake et al. reported that 23.5% of the 51 AN patients attending a clinic (mean age: 27 years) were found to be in the precontemplation stage [19]. Watson et al. reported that 83.4% of patients admitted to a tertiary care university hospital over seven years did so for voluntary treatment (N = 331), while 16.6% were legally committed for involuntary treatment (N = 66) [20]. Thus, a collaborative approach rather than a hierarchical approach was suggested. This relates to the patient as a partner in the management of eating disorders, and is the heart of the described model.

### Collaborative approach

The complexity of EDs calls for a collaborative approach by a multidisciplinary team of mental health, nutrition, and medical specialists [21]. Since co-morbidity for patients with EDs is the rule rather than the exception, recommendations emphasize the importance of specialized care for the treatment of EDs, as well as an intervention model that approaches illnesses from a transdiagnostic orientation, which addresses the dynamics and needs of co-morbidities while treating the ED effectively [21,22]. Pluralism, consumerism, mobility, and increasing access to news, entertainment, and other features of the post-modern world have suggested multiple therapeutic approaches such as narrative therapy and motivational interviewing. Those modalities are currently nested within traditional approaches (psychodynamic and interpersonal psychotherapy, dialectical behavioral therapy, cognitive behavioral therapy (CBT) and family therapy) creating an integrative approach.

Most experts agree that, outpatient family therapy and CBT are a first-line treatments for patients with EDs whose duration of illness has been brief. Severe eating disorders often require a comprehensive and longer intervention [1,22,23]. Fairburn et al. [24] reported that a transdiagnostic CBT appeared to be suitable for the majority of outpatients with an ED. They found that patients with complex psychopathology responded better to CBT that addressed the following maintaining factors: marked mood intolerance, clinical perfectionism, low self-esteem, or interpersonal difficulties. Our treatment model focuses on the second group which often suffers from enduring illness. As Vanderlinden suggested, “treatment can – in some cases – be better and more effectively planned by analysing in more detail the psychopathological or developmental pathway into the eating disorder.” This also holds true in cases that fit well within a transdiagnostic view on eating disorders [25].

Such an integrative approach (Additional file 1: Table S1) has been used in our organization for more than 15 years for patients with severe and enduring illness. We have followed 645 patients with severe eating disorders (258 with anorexia nervosa and 387 with bulimia nervosa), most of whom entered the program when facing difficulties in maintaining regular functioning. Fewer than 10% of patients dropped out during the first two months of treatment (12% among patients diagnosed with BN and 8% among those with AN). Treatment outcomes were assessed using a Global Clinical Score based on the Average Outcome Score developed by Garfinkel et al. [26]. Using this scale remission is when weight is maintained at 15% of ideal body weight, menstruation is regular for at least 12 months purging behaviors are absent, eating habits are normalized and there is good social adjustment as evidenced by being employed or getting back to school. Research assistants blind to the treatment condition administered pre and post intervention measures.

Treatment duration ranged between 15 months to four years. At the end of treatment 69% of those diagnosed with AN and 81%of those diagnosed with BN were in a fully recovered state or much improved. “Fully recovered” was defined as full remission lasting more than 12 months. “Much improved” was defined as partial remission, with infrequent occurrence of the symptoms as well as full occupational and social functioning. All the recovered patients terminated the treatment with mutual consent. Those who were much improved declined regular care and only attended follow-up sessions.

Patients were followed up four year following end of treatment. At this time 68% of those treated with AN and 8% of those treated with BN were categorized as fully recovered or much improved. All patients who completed the program were gainfully employed [27,28].

As described above, the model was used with severely ill patients who had failed to achieve improvement with previous interventions provided by ED clinics and ho had an illness duration between 6 and7 years. Demographic and clinical features are described in Table 1. Most has functional difficulties due to the eating disorder and the goals of treatment were regaining self-agency and developing a cohesive self.

**Table 1:** Baseline demographic and clinical features of subjects (Mean ± SD)

	**Bulimia nervosa**	**Anorexia nervosa**
	**(Mean ± SD)**	**(Mean ± SD)**
	**n = 387**	**n = 258**
**Demographic features**		
Age (yrs), **range:** 11-40	**25.5** ± 5.1	**21.0** ± 4.5
Duration of Illness (yr.)	6.54 ± 6.5	5.7 ± 3.2
**Previous treatments**		
Hospitalization (days)	16.5 ± 34	90.5 ± 112
	(median 2)	(median 45)
	Range 0-150	Range 0-330
Previous out-patient facility specializing in ED (months)	11 ± 4.2	5.6 ± 3.1
	(median 8)	(median 5)
	Range 3-18	Range 1-11
**Symptoms**		
Body mass index	**22.48** ± 3.2	**16.5** ± 4.9
Absent of menses (% of patients)	_	92%
Binge eating (% of patients)	100%	50%
Vomiting (% of patients)	95%	55%
Laxative abuse (% of patients)	**9.3%**	_
**Co-morbidity**		
Post-Traumatic Stress Disorder (% of patients)	4%	1%
Personality disorders/multi impulsivity (% of patients)	**58%**	**34%**

This present paper focuses on the process engaged by a multidisciplinary team in the journey from opposition to change to recovery from eating disorders, employed with these severe patients. It provides a description of the clinical model developed for the multidisciplinary work when treating eating disorders proceeded by a short summary of the mantra and major skills used in narrative therapy and motivational interviewing. Both techniques are integrated within traditional approaches.

### Integrating narrative counseling and motivational interviewing in traditional approaches

In contrast to some past approaches, which relied on authority and a relationship of power, narrative and motivational counseling share a reliance on the clients’ personal agency [29-32]. Both encourage *collaboration*, *evocation*, *autonomy, and compassion,* while empowering patients and striving to help them achieve what is important for them, and thus, to avoid behaviors that place them at risk. The two main practices used to achieve it are externalization in narrative therapy [32];and OARS (Open ended questions, affirmations, reflections and summarizing) in motivational interviewing (MI) [33].

“The problem is the problem, the person is not the problem” is an oft-quoted maxim of Narrative Therapy. The linguistic practice of externalization [29,34] which separates persons from problems, is a playful engaging way to motivate people to face and diminish difficulties. Separating the problem from the person in an externalizing conversation which relieves the pressure of blame and defensiveness.

In order to avoid conflict, resist a righteous stance, and understand clients’ motivations in motivational interviewing, the therapist uses open-ended questions, affirmations, reflections, and summarizing to “dance” with ambivalence rather than confront clients [32]. Williams et al. [35] also incorporate motivational interviewing stance in their multidisciplinary community outreach partnership program to develop and maintain a strong working alliance such that treatment is tailored to the client’s stage of change.

Both the narrative approach and motivational interviewing focus on change. In motivational interviewing, enhancing self-change talk is a core component. The therapist strategically elicits change talk and consistently responds to it when offered. In narrative therapy, a counselor usually asks, “Where does this knowledge take you? In what way will you respond differently now that you have this knowledge?” The therapist is constantly engaged in looking for “unique outcomes”-exceptions to the problem that would not be predicted by the problem’s narrative or story itself and actually assist in creating a preferred story. The idea of “marrying” narrative counseling and motivation interviewing in the treatment of EDs is not unique to our model [36].

Moreover, others have described a format for a change in ED employing a multidisciplinary team referred as developmental-systemic-feminist therapy, or individual developmental-systemic therapy [37]. Bryant-Waugh has provided a structured model for treatment which was also developed in the context of working with people with EDs and their families. It employs five steps to achieve change (explore; understand; accept; challenge; change). However, these steps are termed and described from a more traditional therapy-oriented approach, although the mantra is very similar – both refer to the treatment as a “joint task”.

Our model was developed firstly to assist patients in understanding the different segments of the process while reflecting the development in the patient pathology and acknowledging the need to resist not just the ED but also the “sick role”. The patient feels protected in the status of being ill, and not only under the ED umbrella. We turn the patients into clients. Secondly, our model provides practitioners with an outline of treatment as well as the appropriate skills when integrating narrative and motivational interviewing counseling. At the same time, it explores the origins and evolution of the eating disorder over time as was suggested by Vanderlinden (25).

Our approach is innovative in that it presents a multilevel, multidisciplinary model that goes beyond that of Prochaska and Di Clemente’s [38]. Like many other models, it was developed in response to parents expression of their longing for a sense of control and location while battling their child’s ED.

As a guide map, this model includes five phases along the journey to recovery. We are aware of the risk of over-simplification of an often complex reality when establishing therapeutic models, due to the desire for certainty and comfort, as well as the risk of falling into the trap of “knowing.” We are also aware of the spiral, as opposed to linear, nature of the change process.

**Figure 1:**
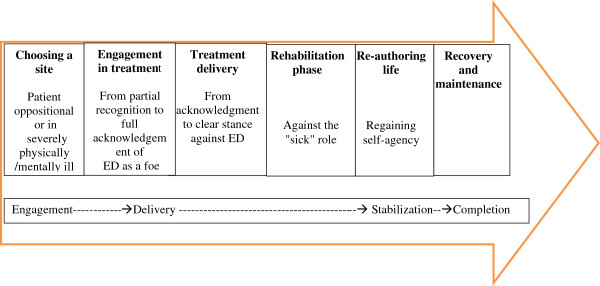
The client journey.

This model offers another view and thus extends the literature rather than replace those views that already exist (Figure 1).

### Eating disorders and the interdisciplinary team

The key to providing quality care for people with EDs and co-morbidities is to coordinate the effort and recommendations of each member of the multidisciplinary team of mental health, nutrition, and medical specialists, who are often unilaterally involved in the initial evaluation and subsequent treatment. This is done with a collaborative approach [1,39,40]. Such multidisciplinary networks may vary by healthcare setting and/or by country. However, the use of a multidisciplinary approach allows diagnostic conclusions and the subsequent plan for treatment to reflect the input and collaboration of the multiple disciplines and centralization of documentation related to the initial evaluation and treatment reports.

Such coordinated multidisciplinary care can influence the course of EDs. The way in which physical and mental health services work together is arguably one of the most important elements of effective care. To that end, a shared understanding of EDs is essential [41].

This manuscript will describe the functional roles of each of the disciplines in the proposed model and the security network provided that is tailored to the needs of patients who are affected by EDs, as well as their families. Since we view emotional dysregulation as the core problem in EDs, each patient is allocated at least one type of therapy aimed at improving his/her mood regulation skills and enhancing an improved emotional states (psychotherapy, art therapy, drama therapy, or biofeedback). Included in therapy are “meaning making” as acceptance and change; active validating of the worth of the individual; and mindfulness skills intended to substitute sensual activities for food satiety.

Parents are invited to participate in a psycho-educational support group where they receive information and emotional support from the group facilitators and other parents who share experiences and offer possible solutions. Nutrition counseling, family therapy, and other components of psychiatric management for patients with eating disorders are also important. Also central to the program are therapeutic alliance, coordinated care and collaboration with other clinicians, assessment and monitoring of symptoms and behaviors, and cognitive and behavioral techniques such as stimulus control procedures and strategies aimed at modifying rigid all-or-nothing thinking and perfectionism.

In addition, during the course of an intensive treatment, five % of patients received between 6 to 12 hours per week of contact with clinical mentors. Clinical mentors were social workers, clinical dietitians, or graduate-level psychology students who were trained to connect with clients in an intensive, informal manner. Senior clinical psychologists supervised them once a week, individually and in a group. The mentors addressed the need for a holding and containing environment, as well as the presence of a strong and reliable emotional resource, countering the ED voice and helping the patient voice his/her own values. They served as meal companions and soothing figures, representing a healthy self-caring image, which countered maladapted patterns of interaction, cognition, and behavior. Social skills training as well as leisure-time activities were encouraged [28].

### The process in the journey from patient’s opposition to change to recovery

The client journey is demonstrated in Figure 1.

#### Preliminary phase

In the preliminary phase, the patient and his/her family underwent an initial evaluation visit that allowed both patient and institute to assess whether the mutual approach fits. This session was first of all an engagement process, aimed at getting to know the client, his/her problem, and how the problem took over his/her life. Ordinarily, this session provided illusionary protection: regulatory issues, fears, desires, self-control issues, social difficulties, personality traits, family conflicts, and the presence of a defensive style (tendency to deny or avoid conflicts). In this session, externalizing conversation was used to position the illness outside the patient and to contradict most patients’ perceptions that they themselves are the problem. During this conversation the patient started to understand how the ED and his/her emotional issues were related. We gently unfolded the development of the signs and symptoms of the ED and taught the patient to identify traces of the ED by him/herself. Some understood, and some did not. Some saw and then forgot. We then asked the patient’s permission to invite his/her parents into the room and to share with them the story of how we (s/he and I) understood the problem to have developed.

We *re-told* the story of the problem, how it invaded the family life, and of which conflicts it took advantage. The patient was then asked to describe why the price had become too high for him/her and what his/her goals were. What were his/her aspirations/life objectives?

•The different budgets that our facility offers were presented, and, with the family, we considered which level of intensity is appropriate for the patient’s physical condition, occupational functioning, and other factors. When a patient felt understood and coul trust the treatment provider, s/he was more ready to enter a meaningful bond and consider treatment as an option.

•After the parents approved the reframed history of the problem, we *set the treatment goals*. The goal of the preliminary phase was to engage the patient and parents in an externalizing conversation in order to reveal the prices and motivate the patient to take control of his/her life and fight the disease. This was achieved using a dynamic understanding, motivational interviewing, and narrative practices and ethos.

##### Phase 1: From partial recognition to full acknowledgment

The first phase of the journey was dedicated to mutual acquaintanceship with the patient, with his/her illness, with his/her family, and most of all, effective practices to promote engagement and evoke internal motivation for change.

Most patients started the program with some level of opposition to change or at least perceived disconnect between life difficulties and inconveniencies and the illness’ impact. Thus, all team members focused on establishing a therapeutic contract and externalizing the ED’s impact on the person’s life.

The dietitian assessed the impact of ED on patients’ eating habits and physical fitness, recognizing the illness’ aims and illusions while assisting the patient to cope with the re-feeding or normalization of eating habits. We took a clear stance against the problem with a firm focus on behavioral goals. We announced clear boundaries and rules, including stages of independence and discussion about which decisions should be mutual and which should not be mutual in each stage. Staff were instructed to clearly communicate that they were not seeking to engage in control battles and were not trying to punish patients with aversive techniques. The role of the patient in the treatment process was equally important to that of the treatment team. In this sense, the patient was an active participant and was accountable for his/her actions in the quest for behavior change and improvement in his/her quality of life.

The psychotherapist focused on the ED’s impact on autonomy, life, wishes, and achievements. This included a discussion of what has been taken from the patients, while in family therapy the focus was on what has been taken from the family space by the ED.

Some personalized programs included clinical mentors. These, provided meal companions and soothing figures, representing the healthy self-caring image. They used narrative and motivational interviewing practices to counter maladapted patterns of interaction, cognition, and behavior as means to foster patients’ hostility towards the illness.

In all types of therapies, the process continued with recognizing the ways in which the ED had taken over patients’ lives (e.g., via isolation, physical and emotional disappearance, engagement in self-policing, empty/false promises, etc.) The process named the current relationships with the problem, and expressed curiosity about how life might look without the ED. Which of their values did the ED attempt to separate them from? In both motivational interviewing and narrative therapy, we sought to develop a distinction between the patient’s current maladaptive state and a more adaptive alternative.

In this way the therapists enlisted patients to form a coalition against the illness, to regain freedom, engaging in change rather than guilt or blame - often the dominant feelings among patients with EDs [42].

##### Phase 2: from acknowledgment to clear cognitive stance against the ED

At this stage most patients followed their trusted figures (parents and therapists), still not having their own internal motivation to recover. Patients perceived the illness as harmful but, at the same time, as a necessary defense strategy against fatness, and thus “agreed” to progress yet were still not “wanting” to progress. During this stage, in all treatment areas, we focused on mapping out anti- and pro-ED steps.

The dietitian continued to externalize and map anti- and pro-ED thinking and behaviors related to foods and physical activity, expanding the patient’s influence on the problem by using reflection and amplification of unique outcomes [42-44]. (Unique outcomes were exceptions to the problem, events in which the patient’s behavior could not be predicted by the problem, and events where patients succeeded in resisting the ED’s temptations). Together they discussed learning alternative options to obtaining a good physical feeling and using cognitive behavioral techniques to enhance performance. Responsibility around food was between the patient and family member or mentor in the various domains (food, social, personal, family) and explores how the person is influencing the problem.

In family therapy, we identified the familial dynamics that enabled the ED to originally set in. These included the influence of culture, ethnicity, discourses, sensitivity, guilt, secrets, strangeness, splits, power relations, family structure, family communication, and values important to the family. We also generated a list of pro- and anti-ED steps taken by the family while affirming its stand against the ED, empowering the relationship they gain back with their child.

Psychotherapists and clinical mentors also expanded the patient’s influence on the problem by using reflection and amplification of unique outcomes to generate a broad description of the skills and knowledge they were drawing on at the time. The next step was to plot an alternative narrative about his/her life and identity.

##### Phase 3: against the “patient” status – delayed adolescence and rehabilitation phase

Most patients entered this stage feeling hostility towards the ED but still favoring the status of being ill. Some described it as a “release from responsibilities, expectations, etc.,” and some said that it gave them a sense of control in their lives. Thus, in this stage, counseling focused on enhancing self-efficacy and supporting patients’ independence.

This phase lasted as long as it took the body and psyche to “catch up” to the developmental stage of body and mind before the ED began. This might be seen as delayed maturation.

The dietitian focus was on expansion of options around physical goals. Independence and increased flexibility around food is encouraged, along with questioning what is best for the patient and what is important for him/her when noticing rigidity around food issues.

The psychotherapist monitored progress in self-regulation, self-control, and coping skills. Self-identity, values, wishes, events, and other developmental steps achieved during the struggle against the illness are recognized. This was done mainly by paying attention to *unique outcomes* (narrative practice), naming preferred stories of rebellion against the ED dictatorship and re-authoring the story of individual identity, according to what is explored in each session. They also explored the patient’s importance, confidence, and ability [45] to move beyond the “sick” status and use a series of “miracle questions” drawn from solution-focused therapy [46] to engage the patient in imagining how his/her life might look if s/he were recovered, independent, and running his/her own life, or how life might look when his/her dreams come true.

In family therapy the focus was around identifying and expanding options towards resisting the illness in general in the family surroundings. This was accomplished by monitoring unique outcomes that expressed stories of stronger familial coping skills and ways of communicating effectively rather than avoidance.

Clinical mentors practiced new experiences around eating with patients, having fun and looking for occupations. When patients perceive themselves as almost normal, mature adolescents, they parted from the clinical mentors.

##### Phase 4: re-authoring life – stabilization and regaining self-agency

In this phase of the ED journey, it seemed that the patient can live without symptoms but would rather not declare this yet.

With the dietitian, conversations focused mainly on assessing what works and monitoring progress towards normal eating behavior. The decisions were transferred to the patient as long as s/he manages to execute them independently from involvement in the ED. The role of the dietitian was increasingly as a “witness” to the changes. Treatment intensity was gradually reduced, and counseling focused on empowering the patient and establishing outsider-witness practices.

The focus of psychotherapies was to rewrite a new narrative in relation to who the patient was; what s/he wants; and how to manage without an ED. Patients were given the option of telling/performing the stories of their lives before an audience of outsider witnesses [42]. The outsider validated his/her response by retelling certain aspects of what has been heard. In EDs practice, the patient was interviewed in front of her/his parents, who served as outsider witnesses and reflected on what they have heard and how the values expressed in what they have heard were actually expressed previously in past stations of the patient’s history. This empowered the patient’s self-agency around the image that was drawn in the room.

##### Phase 5: recovery and maintenance

The patient’s eating behaviors, weight status, and other areas of life become almost normal and stable. Treatment intensity was low. Relapse prevention strategies ware practiced, and a follow-up plan is established. This stage varies in its continuity from patient to patient. It was finalized with a structured farewell passage that includes summation of the patient’s file: each therapist gathered the specific journey process with his/her patient in a way that they chose.

## Conclusion

Narrative and Motivational counseling can operate beyond the standard margins of therapy by situating the “victims” of the problem (both patient and family) in a position of power with respect to the problem and by their therapeutic stance. The therapists are viewed as influential rather than central, helping the patient and family, with little difference in power among them. The therapists work collaboratively, thereby increasing the patient’s and family’s ownership of treatment and outcome and strengthening the therapeutic bond. Both the narrative approach and motivational interviewing share reliance on patients’ personal agency rather than on a relationship of power. Both encourage *collaboration, evocation,* and *autonomy*, as well as compassion, empowerment, and encouragement towards actualization of their self-identified values.

## Competing interest

The author declares no competing interest.

## Supplementary Material

Additional file 1: Table S1 Stages of Engagement and Recuperation from Eating Disorders. Click here for file
