# Supplementary material for: The journey from opposition to recovery from eating disorders: multidisciplinary model integrating narrative counseling and motivational interviewing in traditional approaches
Source: J Eat Disord. 2013 Jun 6;1:19. doi: 10.1186/2050-2974-1-19 (PMC4081798; doi:10.1186/2050-2974-1-19)
Supplement: Additional file 1: Table S1 — Stages of Engagement and Recuperation from Eating Disorders. [file 2050-2974-1-19-S1.docx]

Table S1: Stages of Engagement and Recuperation from Eating Disorders (ED)

| Clinical Mentors | | Family Therapist | | Psychotherapist | | Dietitian Role | Stance against ED | STAGE |
| --- | --- | --- | --- | --- | --- | --- | --- | --- |
| Treatment focus: | | Treatment focus: | | Treatment focus: | | Treatment focus: | The 'melody' |  |
| Preliminary intake session with professional manager, to understand collaboratively, how the problem (using externalizing language) has come into the person’s life. What and how the ED took from as a way to assist the client and his/her family in considering the treatment plan offered. | | | | | | | Frequent denial  of ED | Choosing  site |
| - Therapeutic contract - Common goal, mutual   expectations   - Meal companions and   soothing figures, representing the healthy self-caring image, which counters maladapted  patterns of interaction,  cognition, and behaviors   - Focus on MI strategies   to foster patients’  hostility towards the  illness   - **Emphasizing the**   **creation of coalition to**  **achieve patients’ aims**   - **Reflecting** pro- and   anti-ED behaviors | - Parents only:   Psycho-education to  foster recognition of  illness’ signs, illusions,  traps, and anti-vs. pro-ED parenting style   - Parents + patient: - Exploring losses in   the family space due  to ED   - Assessment of family   dynamics   - Therapeutic contract - Familiarity with   parental strength and  needs at current crises   - Familiarity with family   loses due to ED –  design treatment  goals around important  values. | | - Psychological   assessment   - Engaging patient with   ED’s impacts on  autonomy, life, wishes,  and achievements   - Therapeutic alliance:   goals, mutual  expectations   - **Unmasking ED’s**   **intentions & tactics**   - **Naming the current**   **Relationships with**  **the problem & future**  **wishes about it**   - **Exploring patient’s**   **wishes beyond ED**  **(longings?)**   - Recognizing the   Undesired part of ED | | - Nutritional assessment - Engaging patient with   ED’s impacts   - Therapeutic alliance - **Externalization of ED**   -Recognizing illness  aims, tactics, illusions,  prices, and advantages   - **Exploring eating issues**   - Existing vs. desired  relationship with food  - Coping with re-feeding  and/or normalization  of eating habits   - Negotiation about   eating responsibility | | Despite  Acknowledging the  burden of disease,  it is still an essential  defense  Partial recognition    Hostility towards ED  Following  Acknowledging its  impact (ED still  perceived as a defense  against obesity) | 1  From partial  recognition to  full  acknowledgment |
| - **Continued**   **externalization**   - **Monitoring anti-ED**   **voice and behaviors**   - **Forming strong**   **coalition** reflecting in  real time how the  disease harms the  patient’s emotional,  physical, cognitive,  functional, operational,  social, and familial  functioning   - Assist patient to   overcome pro-AN/BN  thoughts and behaviors via reflection on discrepancies,  empowerment,  and affirmations. | - **Mapping out anti-and**   **pro-AN/BN** behaviors  in the family   - **Recognizing familial**   **dynamics that enabled**  **ED penetration:**   - **i**nfluence of culture, - ethnicity, discourses, - sensitivity, guilt, - secrets, strangeness, - splits, power relations,   family structure, family  communication, values  important to the family   - **Exploring ways and**   **unique outcomes** in  which family resisted  the dominant story  and performed  anti-ED steps | | - **Mapping out anti-**   **and pro-AN/BN**  **thoughts and behaviors**  **that are not related**  **to food.**   - - - - **Recognizing elements**   **that enabled ED**  **penetration and control:** trait and personal elements, social  status, cultural and  familial factors   - **Mapping out thoughts**   **and** behaviors for and  against ED in relation to  emotional difficulties   - **Working towards**   **forming committed**  **stance against ED**   - **Exploring ways and**   **unique outcomes** in  which clients  performed anti-ED steps. | | - Continued **externalization**   **Mapping out anti- and** **pro-**  **AN/BN thoughts and**  **behaviors related to food**   - **Expanding patients’**   influence on the problem  using reflection and  amplification of **unique**  **outcomes-** events where  patients succeeded to resist  ED’s temptations; learning  alternative options to obtain  a good physical feeling as  well as **CBT** strategies to  enhance performance   - R**esponsibility around**   **food is between the**  **patients and family**  **member or mentor.** | | Joining the coalition  Against ED, but  there is still  ambivalence  So:  **Patients**: The disease  is a necessary evil  **Parents:** empathic  to patients’ suffering,  less frustrated  Clear stance  against ED | 2.  2  2.  From  acknowledgment  to clear  cognitive stance  against ED  Guiding towards  a turning point |
| - Focus on the new occupations,   relationships, and  identity formed     - **Mentor moves to a passive** - **place** | - **Identification and**   **expansion of options**  **towards resistant**  **stance against feeding**  **ED** in particular and  illness in general in  family surroundings   - Monitoring unique   outcomes, rewriting  richer familial narrative  Story of stronger  familial identity, wishes,  and demeanors.  Emphasizing the new  continual demeanor in  order to essentially  create a new narrative  and not a series of  sentences | | - - - - **Monitoring progress** in   Coping skills recognition  of self –identity values,  wishes, events, and other  developmental steps  achieved during the  struggle against illness  done mainly via  attention to **unique**  **outcomes** and the  strength and value behind  these events   - Selective reflections on   advantages of healthy  status and of self-care   - Discussing general   coping skills, handling  conflicts, social skills,  self-regulation, and  self-control issues, as  well as other conflicts. | | - - - - Exploring options around   physical goals   - Independence around   food is encouraged   - The dietitian’s role is   gradually transferring to  witness role reflecting  the changes that clients  experience in relation to  different area of life  following freedom from ED   - Using guiding style   when noticing stiffness  around food issues | | Change in  perceptions around  ED, but yet not  around being sick  Rehabilitation  Clear stance  against being sick | 3  Against the  “patient” status  Rehabilitation  phase |
| **Parting from mentors**  **identity**  Client perceived as  If almost normal  mature adolescent or  adult | **Rewriting of the**  **familial story**  **identity**  Glorification of  unique event and  what it means in  relation to the new  family identity | | - **Rewriting a new**   **narrative** in relation  to who I am, what I  want, how I manage  without an eating  disorder, how I handle  the situation.   - Exploring new ways   to cope with these  issues and why  patients can be assured  that ED has no more  reason to come into  their life | | - **A new narrative in**   **relation to food**:   - Focus on normalization   of eating behavior   - Only when the choices   are independent and  without the intervention  of the disorder are the  decisions are transferred to  patients; counseling and  the **dietician take the**  **role of "witnesses"**  **to the changes** | | **Can live without**  **the symptoms but**  **would rather not**  **declare it yet**  (difficulty to  relinquish patients’  identity-treatment  should continue)  Creating identity of healthy person  Re-authoring life | 4  Re-authoring life  Regaining self  agency |
|  | **Dealing with farewells**  In the family, developing  the attitude towards  separation as part of a  process in which one  door closes and another  one just opens, as a  growth step, as a source  of pride to parents. | | - Treatment becomes   overbearing -request  for lowering intensity   - Dealing with   mourning over the  years that were "lost"  and parting   - Discussing separation   issues. | | - **Practicing normalization**   with eating behaviors  and stabilizing long term  weight status   - **Relapse prevention**   **plan** | | I am post-ED,  feel healthy  Wants to check if I  can stay with  a “bit” of disease  COVERY | 5  Recovery and  maintenance |
